# The TERT rs2736100 Polymorphism and Cancer Risk: A Meta-analysis Based on 25 Case-Control Studies

**DOI:** 10.1186/1471-2407-12-7

**Published:** 2012-01-05

**Authors:** Peng Zou, Aihua Gu, Guixiang Ji, Lin Zhao, Peng Zhao, Ailin Lu

**Affiliations:** 1 Department of neurosurgery, the First Affiliated Hospital of Nanjing Medical University, Nanjing, 210029, China; 2 School of Public Health, Nanjing Medical University, Nanjing 210029, China; 3 Nanjing Institute of Environmental Sciences/Key Laboratory of Pesticide Environmental Assessment and Pollution Control, Ministry of Environmental Protection, Nanjing 210042, China

## Abstract

**Background:**

The association between the *TERT rs2736100 *single nucleotide polymorphism (SNP) and cancer risk has been studied by many researchers, but the results remain inconclusive. To further explore this association, we performed a meta-analysis.

**Methods:**

A computerized search of PubMed and Embase database for publications on the *TERT rs2736100 *polymorphism and cancer risk was performed and the genotype data were analyzed in a meta-analysis. Odds ratios (ORs) with 95% confidence intervals (CIs) were estimated to assess the association. Sensitivity analysis, test of heterogeneity, cumulative meta-analysis and assessment of bias were performed in our meta-analysis.

**Results:**

A significant association between the *TERT rs2736100 *polymorphism and cancer susceptibility was revealed by the results of the meta-analysis of the 25 case-control studies (GG versus TT: OR = 1.72, 95% CI: 1.58, 1.88; GT versus TT: OR = 1.38, 95% CI: 1.29, 1.47; dominant model-TG + GG versus TT: OR = 1.47, 95% CI: 1.37, 1.58; recessive model-GG versus TT + TG: OR = 1.37, 95% CI 1.31, 1.43; additive model-2GG + TG versus 2TT + TG: OR = 1.30, 95% CI: 1.25, 1.36). Moreover, increased cancer risk in all genetic models was found after stratification of the SNP data by cancer type, ethnicity and source of controls.

**Conclusions:**

In all genetic models, the association between the *TERT rs2736100 *polymorphism and cancer risk was significant. This meta-analysis suggests that the *TERT rs2736100 *polymorphism may be a risk factor for cancer. Further functional studies between this polymorphism and cancer risk are warranted.

## Background

Cancer is a multifactorial disease, which is the result of complex interactions between inherited and environmental factors. Lung cancer is the most common malignancy and the leading cause of cancer deaths for women and men worldwide [1-3]. There are two main histologic subgroups of lung cancer: small-cell lung carcinoma (SCLC) and non-small-cell lung carcinoma (NSCLC); the latter includes the common types, which are squamous cell carcinoma (SCC) and adenocarcinoma (ADC). Gliomas of astrocytic, oligodendroglial, and ependymal origin are derived from glial cells and account for Fax~80% of malignant primary brain tumors (PBTs), which are the most common histologic type of brain tumors [4]. There is a dose-response relationship between ionizing radiation and the risk of developing an intracranial tumor [5], whereas familial aggregation of gliomas [6] is a result of a combination of low-risk variants.

Telomeres are special nucleoprotein structures located at the ends of eukaryotic chromosomes and are essential for protecting chromosomal termini against degradation, end to-end fusion and rearrangement [7]. Telomeres are composed of repetitive DNA (TTAGGG repeats) bound to abundant specialized proteins. The length of telomere repeats as well as the integrity of telomere-binding proteins are essential for telomere maintenance [8]. Telomerase recognizes the 3' hydroxyl (3' OH) at the end of the G-strand overhang and adds telomeric repeat sequences onto chromosome ends. Telomerase expression can prevent telomere erosion in most eukaryotic organisms. Functional telomerase is composed of the TERT (telomerase reverse transcriptase) protein and the telomerase RNA component (TERC) that acts as a template for DNA synthesis. In contrast to TERC, which is expressed rather ubiquitously, TERT expression is low in most normal human somatic tissues and is physiologically restricted to primary germ line cells, tissue stem cells and activated lymphocytes [9-14], leading researchers to consider TERT as the limiting factor for telomerase activity. The *TERT *gene product contains three distinct structural domains: the RNA-binding domain (TRBD), the reverse transcriptase domain and the carboxy-terminal extension (CTE), which represents the putative thumb domain of TERT [15]. Tumor cells can prevent telomere loss through the abnormal upregulation of telomerase [16], and telomerase has been found to be reactivated in the majority of cancers, including those of the lung [7]. Activation of telomerase induced by the catalytic component TERT is a pivotal step during cellular immortalization and malignant transformation of human cells [17].

In the past decade, many investigators have explored factors contributing to inherited susceptibility to cancer [18]. The sequence variants in the *TERT *and *cleft lip and palate transmembrane 1 like *(*CLPTM1L*) gene regions are associated with susceptibility to many types of cancer [19]. The *rs2736100 *polymorphism is localized to intron 2 of the *TERT *gene. McKay et al. [20] published the first study indicating that the *TERT rs2736100 *polymorphism may contribute to an increased risk of lung cancer. Since then, several research groups have reported associations between this SNP and cancer risk, but with inconclusive results [21-31]. Consequently, we performed a meta-analysis to more precisely characterize this association.

## Methods

### Study eligibility and identification

Eligible studies were identified by searching PubMed, Embase, CNKI, and the Chinese Biomedicine Database (the last search update was performed on November 15, 2011), using the following search terms (*TERT *OR "telomerase reverse transcriptase") AND polymorphism and using the limits, Humans, English, Cancer. The related reference articles were searched to identify other relevant publications. Unpublished data and further information were also obtained from the authors. The case-control studies were selected if data were available on the role of the *TERT rs2736100 *polymorphism in cancer risk.

In our meta-analysis, the following inclusion criteria were used for selecting the studies: (1) articles about the *TERT rs2736100 *polymorphism and cancer risk, (2) case-control design, and (3) sufficient genotype data for estimating an odds ratio (OR) with a 95% confidence interval (CI). Articles that were not about cancer research, contained duplicated previous research, or did not include usable genotype data were excluded.

### Data extraction

Two investigators independently extracted the data from all eligible publications using the selection criteria listed above. Any disagreement was resolved by discussion. We extracted the following information from each study when available: the first author's name, year of publication, country, patient ethnicity (composed of either European or Asian), cancer type, source of control groups (population- or hospital-based controls or mixed (composed of both population- and hospital-based controls)), genotyping method and number of cases and controls with the TT, TG, and GG genotypes.

### Data synthesis

All statistical analyses were performed using the STATA software (version 11; Stata Corporation, College Station, Texas). Two-sided *P *values less than 0.05 were considered statistically significant. We first assessed Hardy-Weinberg equilibrium in the control groups of each study. The OR and 95% CI in each case-control study were employed to assess the strength of the associations between the *TERT rs2736100 *polymorphisms and cancer risk. The OR and the 95% CI in each comparison were assessed in a codominant model (GG versus TT; GT versus TT), a dominant model (GG + GT versus TT), a recessive model (GG versus GT + TT) and an additive model (2GG + TG versus 2TT + TG). Subgroup analyses were performed based on cancer type, the source of controls and ethnicity. The chi-square test-based *Q*-statistic was calculated to test the heterogeneity between studies. If the result of this heterogeneity test was *P *< 0.05, then the pooled ORs were analyzed using the random effects model (the DerSimonian and Laird method) [32]. Otherwise, if the result of this heterogeneity test was *P *> 0.05, indicating that the between-study heterogeneity was not significant, then the fixed-effects model was selected (the Mantel-Haenszel method) [33]. The *I^2 ^*(*I^2 ^= 100% × (Q-df)/Q*) statistic was then used to quantitatively estimate heterogeneity, with *I^2 ^*<25%, 25-75% and >75% representing low, moderate or high degrees of inconsistency, respectively [34,35]. The significance of the combined OR was determined using the *Z *test (*P *< 0.05 was considered statistically significant). Additionally, sensitivity analyses were performed after sequential removal of each study. Cumulative meta-analyses were performed through an assortment of all eligible cancer studies with case sample size. Finally, the Begg's funnel plot and Egger's test were performed to analyze the publication bias statistically (*P *< 0.05 was considered a significant publication bias) [36].

## Results

### Eligible studies

**Figure 1:**
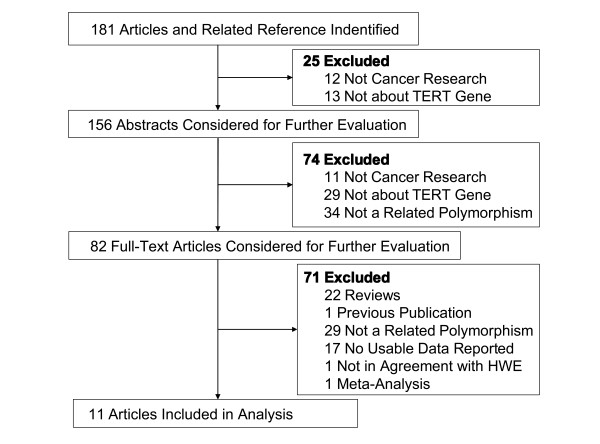
**The study inclusion and exclusion procedures**.

In total, 11 articles including 25 case-control studies in English with 23032 cases and 38274 controls met the inclusion criteria. The characteristics of the studies are listed in Table 1. In our meta-analysis, most of the cancer types were lung cancer and glioma. Among the 25 studies, 14 focused only on lung cancer, 9 focused only on glioma and 2 focused on other cancers. The 25 studies collected in this meta-analysis included 15 studies of Asians and 10 studies of Europeans, 17 studies of population-based controls, 6 studies of hospital-based controls and 2 study of population-based and hospital-based controls. Figure 1 shows the study selection procedure. The main results of this meta-analysis were listed in Table 2.

**Table 1:** Study characteristics from published studies on the relation of the *TERT rs2736100 *polymorphism to cancer risk in this meta- analysis

ID	First author	Year	Ethnicity	Cancer type	Source of controls	Total	Cases			Controls			HWE
							
							TT	TG	GG	TT	TG	GG	
1	Jin(China)[21]	2009	Asian	Lung cancer	PB	2551	353	627	232	450	658	231	0.72
2	Wang(England) [22]	2010	European	Lung cancer	PB	792	42	115	82	136	259	158	0.15
3	Kohno(Japan) [23]	2010	Asian	Lung cancer	MIXED	2624	488	796	372	373	460	135	0.72
4	Hsiung(China-GELAC) [24]	2010	Asian	Lung cancer	PB	1169	118	330	136	225	278	82	0.79
5	Hsiung(China-GELAC(replication)) [24]	2010	Asian	Lung cancer	PB	1170	156	318	136	214	260	86	0.63
6	Hsiung(China-CAMSCH) [24]	2010	Asian	Lung cancer	PB	556	71	122	77	87	154	45	0.09
7	Hsiung(Korea-SNU) [24]	2010	Asian	Lung cancer	PB	542	87	125	44	114	141	31	0.19
8	Hsiung(Korea-KNUH) [24]	2010	Asian	Lung cancer	PB	227	40	52	26	44	46	19	0.25
9	Hsiung(Korea-KUMC) [24]	2010	Asian	Lung cancer	PB	176	31	47	15	31	41	11	0.66
10	Hsiung(China-WHLCS) [24]	2010	Asian	Lung cancer	HB	381	50	83	42	65	104	37	0.68
11	MiKi(Japan) [25]	2010	Asian	Lung cancer	MIXED	2904	291	498	215	696	890	314	0.30
12	MiKi(Japan(replication)) [25]	2010	Asian	Lung cancer	HB	8201	157	273	95	2830	3664	1182	0.94
13	MiKi(Korea) [25]	2010	Asian	Lung cancer	HB	2015	174	277	106	567	692	199	0.60
14	Hu(China) [26]	2011	Asian	Lung cancer	HB	17937	2393	4294	1872	3231	4533	1614	0.72
15	Shete(England) [27]	2009	European	Glioma	PB	2065	115	316	200	349	676	409	0.04
16	Shete(America) [27]	2009	European	Glioma	HB	3480	230	645	372	546	1103	584	0.58
17	Shete(France) [27]	2009	European	Glioma	PB	2913	225	686	441	383	807	371	0.18
18	Shete(German) [27]	2009	European	Glioma	PB	1056	91	240	160	133	269	163	0.28
19	Shete(Sweden) [27]	2009	European	Glioma	PB	1387	120	326	177	212	367	185	0.29
20	Wrensch(America) [28]	2009	European	Glioma	PB	4672	95	354	242	1021	1904	1056	0.01
21	Schoemaker(Denmark) [29]	2010	European	Glioma	PB	265	22	58	39	31	74	41	0.82
22	Schoemaker(Finland) [29]	2010	European	Glioma	PB	192	8	56	33	23	53	19	0.25
23	Chen(China) [30]	2011	Asian	Glioma	HB	1989	244	515	194	334	542	160	0.01
24	Gago-Dominguez(America) [31]	2010	European	Bladder cancer	PB	1018	86	239	146	127	262	158	0.36
25	Gago-Dominguez(China) [31]	2010	Asian	Bladder cancer	PB	1024	141	260	98	174	274	77	0.06

PB: population based; HB: hospital based

**Table 2:** Stratified analyses of the *TERT rs2736100 *polymorphism on cancer risk

Variables	GG versus TT		GT versus TT		Dominant model		Recessive model		Additive model	
	**OR(95%CI)**	***P*^a^**	**OR(95%CI)**	***P*^a^**	**OR(95%CI)**	***P*^a^**	**OR(95%CI)**	***P*^a^**	**OR(95%CI)**	***P*^a^**
Total	1.72(1.58-1.88)^b^	0.002	1.38(1.29-1.47)^b^	0.012	1.47(1.37-1.58)^b^	0.001	1.37(1.31-1.43)	0.062	1.30(1.25-1.36)^b^	0.004
**Cancer type**										
Lung cancer	1.74(1.54-1.96)^b^	0.008	1.34(1.23-1.46)^b^	0.031	1.44(1.32-1.57)^b^	0.009	1.39(1.32-1.47)	0.077	1.32(1.24-1.40)^b^	0.009
Glioma	1.76(1.51-2.06)^b^	0.017	1.47(1.36-1.60)	0.138	1.57(1.39-1.77)^b^	0.040	1.34(1.24-1.44)	0.174	1.31(1.22-1.40)^b^	0.044
Other cancer	1.46(1.13-1.89)	0.592	1.24(1.01-1.54)	0.522	1.30(1.06-1.59)	0.725	1.22(0.99-1.51)	0.245	1.19(1.05-1.34)	0.644
**Source of control**										
Population based	1.80(1.56-2.07)^b^	0.001	1.44(1.28-1.61)^b^	0.004	1.53(1.36-1.73)^b^	0.000	1.38(1.29-1.47)	0.069	1.32(1.24-1.42)^b^	0.002
Hospital based	1.57(1.46-1.68)	0.949	1.29(1.22-1.37)	0.892	1.36(1.29-1.44)	0.963	1.33(1.25-1.41)	0.637	1.25(1.21-1.29)	0.960
**Ethnicity**										
Asian	1.72(1.54-1.92)^b^	0.013	1.32(1.22-1.43)^b^	0.041	1.42(1.31-1.54)^b^	0.013	1.40(1.33-1.47)	0.111	1.31(1.24-1.38)^b^	0.013
European	1.72(1.48-2.01)^b^	0.016	1.50(1.37-1.63)	0.266	1.58(1.46-1.72)	0.077	1.31(1.22-1.41)	0.160	1.30(1.21-1.39)^b^	0.040

^a^*P *value of Q-test for heterogeneity test

^b^Random-effects model was used when *P *value for heterogeneity test <0.05; otherwise, fix-effects model was used

### Evidence synthesis

**Figure 2:**
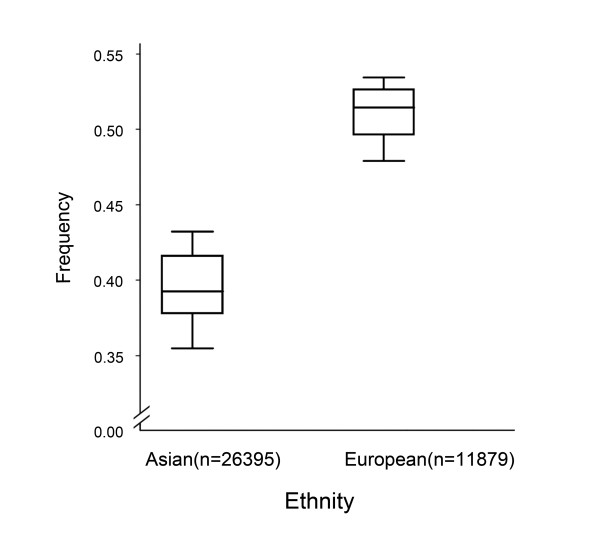
**Frequencies of the variant alleles among controls stratified by ethnicity**.

There was wide variation in the *TERT rs2736100 *polymorphism among the controls across different ethnicities. For European populations, the G allele frequency was 51.0% (95% CI = 49.6-52.4), which was significantly (*P *<0.001) higher than that in the Asian populations (39.6%, 95% CI = 38.4-40.8) (Figure 2).

**Figure 3:**
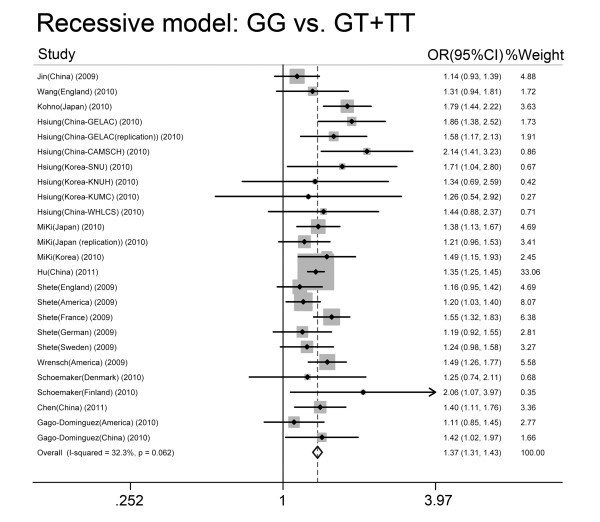
**Overall meta-analysis of the *TERT rs2736100 *polymorphism and cancer risk in the recessive model**.

As shown in Table 2, for the *TERT rs2736100 *polymorphism, all studies combined (23032 cases and 38274 controls) were pooled into the meta-analysis, and a significantly increased cancer risk was found for all genetic models based on the studies (GG versus TT: OR = 1.72, 95% CI: 1.58, 1.88; GT versus TT: OR = 1.38, 95% CI: 1.29, 1.47; dominant model-TG + GG versus TT: OR = 1.47, 95% CI: 1.37, 1.58; recessive model-GG versus TT + TG: OR = 1.37, 95% CI 1.31, 1.43; additive model-2GG + TG versus 2TT + TG: OR = 1.30, 95% CI: 1.25, 1.36). Figure 3 shows the overall meta-analysis of the *TERT rs2736100 *polymorphism and cancer risk in the recessive model.

### Subgroup analysis

Specific data for the *TERT rs2736100 *polymorphism were stratified by cancer type: the lung cancer subgroup, the glioma subgroup and the other cancers subgroup. The pooled odds ratios for the lung cancer, glioma, the other cancers were 1.39 (95% CI 1.32, 1.47), 1.34 (95% CI 1.24, 1.44) and 1.22 (95% CI 0.99, 1.51), respectively, when we use a recessive genetic model. The meta-analysis results for the other genetic models are listed in Table 2.

In the stratified analysis by source of controls, significantly increased risks were also found. The pooled odds ratios were 1.38 (95% CI 1.29, 1.47) in the population-based controls subgroup and 1.33 (95% CI 1.25, 1.41) in the hospital-based controls subgroups in a recessive genetic model. The meta-analysis results for the other genetic models are listed in Table 2.

We stratified the studies by the ethnicity of the participants into two subgroups, Asian and European, and the pooled odds ratios were 1.40 (95% CI 1.33, 1.47) and1.31 (95% CI 1.22, 1.41), respectively, in a recessive genetic model. The meta-analysis results for the other genetic models are listed in Table 2.

Among the 25 studies, many investigators also have established an association between the *TERT rs2736100 *polymorphism and risk of lung adenocarcinoma (Table 3). As shown in Table 4, a significant association was observed between the *TERT rs2736100 *polymorphism and adenocarcinoma susceptibility in all genetic models (GG versus TT: OR = 1.85, 95% CI: 1.72, 1.98; GT versus TT: OR = 1.44, 95% CI: 1.31, 1.59; dominant model-TG + GG versus TT: OR = 1.56, 95% CI 1.42, 1.71; recessive model-GG versus TT + TG: OR = 1.50, 95% CI: 1.41, 1.60; additive model-2GG + TG versus 2TT + TG: OR = 1.36, 95% CI: 1.31, 1.41).

**Table 3:** Study characteristics from published studies on the relation of the *TERT rs2736100 *polymorphism to adenocarcinoma in this meta-analysis

ID	First author	Year	Ethnicity	Cancer type	Source of controls	Total	Cases			Controls			HWE
							
							TT	TG	GG	TT	TG	GG	
1	Jin(China)[21]	2009	Asian	Adenocarcinoma	PB	374	38	101	32	73	98	32	0.93
2	Wang(England)[22]	2010	European	Adenocarcinoma	PB	665	13	60	39	136	259	158	0.15
3	Kohno(Japan)[23]	2010	Asian	Adenocarcinoma	MIXED	2624	488	796	372	373	460	135	0.72
4	Hsiung(China-GELAC)[24]	2010	Asian	Adenocarcinoma	PB	1169	118	330	136	225	278	82	0.79
5	Hsiung(China-GELAC(replication))[24]	2010	Asian	Adenocarcinoma	PB	967	99	213	95	214	260	86	0.63
6	Hsiung(China-CAMSCH)[24]	2010	Asian	Adenocarcinoma	PB	474	47	88	53	87	154	45	0.09
7	Hsiung(Korea-SNU)[24]	2010	Asian	Adenocarcinoma	PB	503	70	109	38	114	141	31	0.19
8	Hsiung(Korea-KNUH)[24]	2010	Asian	Adenocarcinoma	PB	212	35	45	23	44	46	19	0.25
9	Hsiung(Korea-KUMC)[24]	2010	Asian	Adenocarcinoma	PB	149	20	37	9	31	41	11	0.66
10	Hsiung(China-WHLCS)[24]	2010	Asian	Adenocarcinoma	HB	295	19	48	22	65	104	37	0.68
11	MiKi(Japan)[25]	2010	Asian	Adenocarcinoma	MIXED	2904	291	498	215	696	890	314	0.30
12	MiKi(Japan(replication))[25]	2010	Asian	Adenocarcinoma	HB	8201	157	273	95	2830	3664	1182	0.94
13	MiKi(Korea)[25]	2010	Asian	Adenocarcinoma	HB	2015	174	277	106	567	692	199	0.60
14	Hu(China)[26]	2011	Asian	Adenocarcinoma	HB	13701	1148	2155	1020	3231	4533	1614	0.72

PB: population based; HB: hospital based

**Table 4:** Stratified analyses of the *TERT rs2736100 *polymorphism on adenocarcinoma

Variables	GG versus TT		GT versus TT		Dominant model		Recessive model		Additive model	
	**OR(95%CI)**	***P*^a^**	**OR(95%CI)**	***P*^a^**	**OR(95%CI)**	***P*^a^**	**OR(95%CI)**	***P*^a^**	**OR(95%CI)**	***P*^a^**
Total	1.85(1.72-1.98)	0.117	1.44(1.31-1.59)^b^	0.037	1.56(1.42-1.71)^b^	0.030	1.50(1.41-1.60)	0.447	1.36(1.31-1.41)	0.169
**Source of control**										
Population based	2.36(1.96-2.83)	0.515	1.71(1.48-1.98)	0.052	1.86(1.62-2.13)	0.078	1.64(1.41-1.92)	0.648	1.52(1.39-1.65)	0.497
Hospital based	1.74(1.59-1.90)	0.532	1.34(1.24-1.44)	0.954	1.44(1.34-1.54)	0.875	1.45(1.34-1.57)	0.454	1.32(1.26-1.38)	0.610

^a^*P *value of Q-test for heterogeneity test

^b^Random-effects model was used when *P *value for heterogeneity test <0.05; otherwise, fix-effects model was used

### Sensitivity analysis

**Figure 4:**
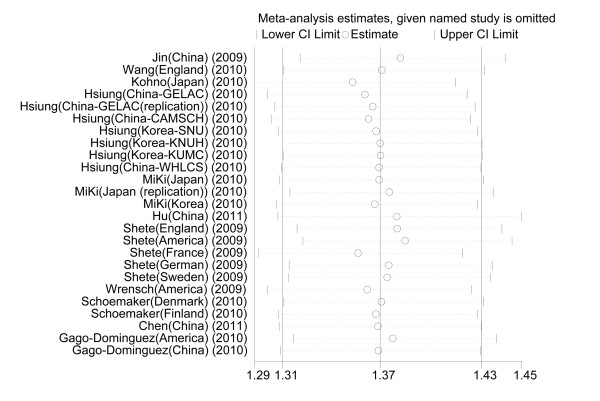
**Influence analysis for GG versus GT/TT in the overall meta-analysis**. This figure shows the influence of individual studies on the summary OR. The middle vertical axis indicates the overall OR and the two vertical axes indicate the 95% CI. Open circles indicate the pooled OR when the study indicated on the left is omitted from the meta-analysis. The lines indicate the 95% CI values when the study indicated is omitted from the meta-analysis.

Sensitivity analyses were performed after sequential removal of each eligible study. When we investigated the *TERT rs2736100 *polymorphism and cancer susceptibility, the results suggested that the significance of the pooled ORs was not influenced by any single study in a recessive genetic model. Sensitivity analyses indicated that the independent study contributing the most to heterogeneity was conducted by Kohno et al. [23] (Figure 4). The heterogeneity was effectively decreased by exclusion of that study: OR = 1.37 (95% CI: 1.31, 1.43; *P *_heterogeneity _= 0.062; *I^2 ^*= 32.3%) and 1.35 (95% CI: 1.29, 1.41; *P *_heterogeneity _= 0.174; *I^2 ^*= 21.2%) before and after removal, respectively.

### Test of heterogeneity

Significant heterogeneity existed in four genetic models (GG versus TT, GT versus TT, TG + GG versus TT, 2GG + TG versus 2TT + TG) of the *TERT rs2736100 *polymorphism (Table 2). However, stratification based on the source of controls reduced the heterogeneity in the hospital-based controls subgroups (GG versus TT: *P *_heterogeneity _= 0.949, *I^2 ^*= 0.0%; GT versus TT: *P *_heterogeneity _= 0.892, *I^2 ^*= 0.0%; TG + GG versus TT: *P *_heterogeneity _= 0.963, *I^2 ^*= 0.0%; 2GG + TG versus 2TT + TG; *P *_heterogeneity _= 0.960, *I^2 ^*= 0.0%). When patients were stratified based on ethnicity, heterogeneity disappeared in the European (GT versus TT: *P *_heterogeneity _= 0.266, *I^2 ^*= 19.2%; TG + GG versus TT: *P *_heterogeneity _= 0.077, *I^2 ^*= 42.1%). In the analysis of the cancer type subgroups, heterogeneity disappeared in the glioma (GT versus TT: *P *_heterogeneity _= 0.138, *I^2 ^*= 35.0%).

### Cumulative meta-analysis

**Figure 5:**
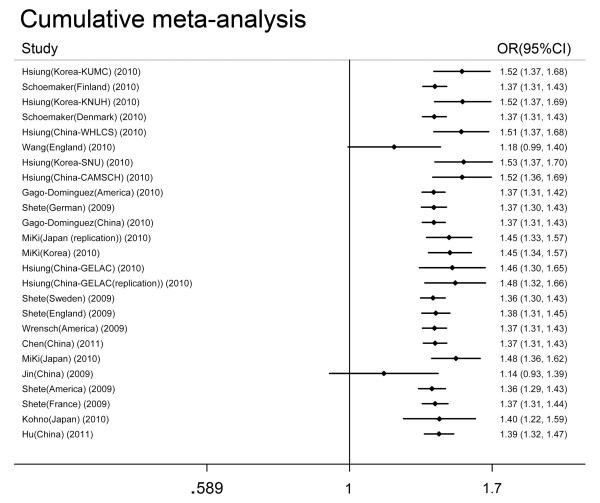
**Results of the cumulative meta-analysis of associations between the *TERT rs2736100 *polymorphism and cancer risk in the recessive model**. The studies were sorted based on case sample size (small to large).

Cumulative meta-analyses were also conducted using the eligible studies sorted by case sample size (Figure 5). There is no obvious change in the 95% confidence intervals with increasing sample size.

### Assessment of bias

**Figure 6:**
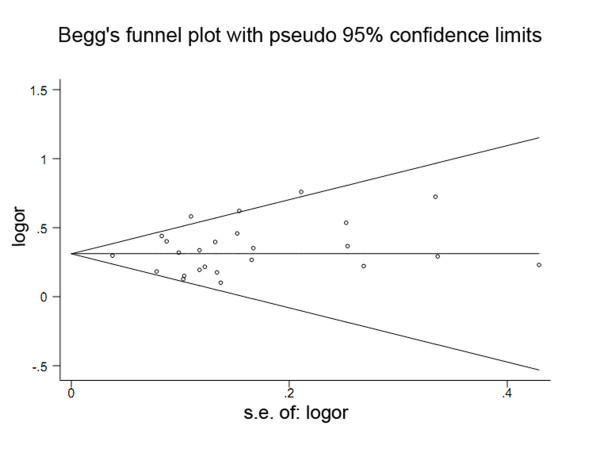
**Funnel plot of the *TERT rs2736100 *polymorphism and cancer risk data for publication bias**.

The Begg's funnel plot and Egger's test were performed to assess the publication bias (Figure 6). The results did not show any evidence of publication bias (*t *= 1.03, *P *= 0.313 for GG versus GT + TT), and the 95% confidence interval (95% CI: -0.49, 1.47) included zero, indicating no publication bias. Additionally, in all genetic models, the results did not show evidence of publication bias.

## Discussion

It is well known that single nucleotide polymorphisms (SNPs) are the most common sources of human genetic variation, which may contribute to an individual's susceptibility to cancer [37]. Thus genetic susceptibility to cancer has been extensively studied in the scientific community. A single nucleotide polymorphism (SNP) *rs2736100 *located in intron 2 of *TERT*, has been hypothesised to be associated with the risk of cancer development by many researchers, however, the results are conflicting and heterogeneous. Here, we analyzed pooled data from case-control studies to determine the role of *TERT rs2736100 *polymorphism in cancer susceptibility. In the meta-analysis conducted on 23032 cases and 38274 controls, *TERT rs2736100 *polymorphism was found to be associated with a significantly increased cancer risk. In the subgroup analyses by cancer type, ethnicity and source of controls, increased cancer risk in all genetic models was also found.

A number of well-designed genome-wide association studies (GWAS) have implicated variants at the 5p15.33 locus (containing the *TERT *gene) in cancer risk at several different sites; lung cancer, basal cell carcinoma and pancreatic cancer show strong associations, while bladder, prostate and cervical cancer as well as glioma show risk alleles in this region [38]. The biology of *TERT *makes it a compelling candidate gene for factors that influence cancer risk [39] and the *TERT *gene has been recognized as one of the most common tumor markers. The *TERT *gene is located on the short (p) arm of chromosome 5 at position 15.33 which is the reverse transcriptase component of telomerase and the expression of the functional TERT protein is a prerequisite for acquisition of telomerase activity. Telomerase is a ribonucleoprotein enzyme that synthesizes the TTAGGG telomeric repeat sequences that are essential for genomic stability [40,41]. Activation of telomerase has been implicated in human cell immortalization and cancer cell pathogenesis and telomerase reexpression is a key factor in cancer cell biology, enabling malignant cells to proliferate indefinitely [7]. The commonly observed high expression of telomerase in lung cancer suggests that *TERT *may have an important role in lung tumorigenesis [7,42-44]. Telomerase activity is present in most glioma samples while absent in normal brain tissues [45]. TERT expression also correlates with glioma grade and prognosis [46,47]. Moreover, the reduction in telomerase activity may inhibit glioma cell growth [48].

Telomerase and the control of telomere length are intimately linked to the process of tumourigenesis in humans [38]. The association between *TERT *polymorphisms (rs401681 and rs2736098) and shorter telomere length has been recently reported [19]. The type of alteration (short vs. long telomere length) linked to a poorer survival might depend on the tumor type [49]. However, the functional significance of the SNP *rs2736100 *was not clear. *TERT rs2736100 *polymorphism may contribute directly to disease predisposition by modifying the function of TERT or is in highly linkage disequilibrium (LD) with other nearby biologically plausible and disease-causing mutations.

Adenocarcinoma is the most common histologic type of lung cancer and the relative proportion of ADC has steadily risen. The strongest risk association was observed between the *TERT rs2736100 *polymorphism and adenocarcinoma in all genetic models. *TERT *gene amplification occurred in 57% of NSCLCs, but was more common among ADCs (75%). *TERT *gene amplification is responsible for *TERT *mRNA overexpression in a majority of ADCs, while epigenetic factors at the transcriptional or post-transcriptional levels significantly affect TERT expression in NSCLC cells [50]. The re-expression of TERT may indicate progression from bronchiolo-alveolar carcinoma to adenocarcinoma [7,51].

There are some limitations of this meta-analysis that should be discussed. First, misclassifications of the histologic type of the cancers reported may influence the results. Second, the lack of detailed information, such as age and sex of the patients, in some studies limited further stratification, and a more accurate OR would be corrected for age, sex and other factors that are associated with cancer risk. Third, in our meta-analysis, the origins of heterogeneity may include many factors, such as the differences in control characteristics and diverse genotyping methods. In addition, the small sample size (<100 cases and controls) studies appear to overestimate the true association because of deficiencies in statistical power.

Based on the limitations of the present study listed above, detailed studies are warranted to confirm our findings. Nevertheless, our meta-analysis has some advantages. First, the well-designed search and selection method significantly increased the statistical power of this meta-analysis. Second, the distribution of genotypes in the controls was consistent with Hardy-Weinberg equilibrium (*P > 0.01*) in all studies. Third, the results did not show any evidence of publication bias.

## Conclusions

The overall results of this meta-analysis have shown that the *TERT rs2736100 *polymorphism is associated with cancer risk. Further functional studies between this polymorphism and cancer risk are warranted.

## Competing interests

The authors declare that they have no competing interests.

## Authors' contributions

PZ participated in collection of data and manuscript preparation. GJ and LZ performed the statistical analysis. PZ and AG participated in study design and critically revised the manuscript. PZ and AL participated in study design and manuscript preparation. All authors read and approved the final manuscript.

## Pre-publication history

The pre-publication history for this paper can be accessed here:

http://www.biomedcentral.com/1471-2407/12/7/prepub
